# The Effects of Vagus Nerve Stimulation on Ventricular Electrophysiology and Nitric Oxide Release in the Rabbit Heart

**DOI:** 10.3389/fphys.2022.867705

**Published:** 2022-06-08

**Authors:** Emily Allen, Pott Pongpaopattanakul, Reshma A. Chauhan, Kieran E. Brack, G. André Ng

**Affiliations:** ^1^ Department of Cardiovascular Sciences, Glenfield Hospital, University of Leicester, Leicester, United Kingdom; ^2^ NIHR Leicester BRC, Glenfield Hospital, Leicester, United Kingdom

**Keywords:** electrophysiology, autonomic nervous system, heart, vagus nerve stimulation, nitric oxide

## Abstract

**Background:** Abnormal autonomic activity including impaired parasympathetic control is a known hallmark of heart failure (HF). Vagus nerve stimulation (VNS) has been shown to reduce the susceptibility of the heart to ventricular fibrillation, however the precise underlying mechanisms are not well understood and the detailed stimulation parameters needed to improve patient outcomes clinically are currently inconclusive.

**Objective:** To investigate NO release and cardiac electrophysiological effects of electrical stimulation of the vagus nerve at varying parameters using the isolated innervated rabbit heart preparation.

**Methods:** The right cervical vagus nerve was electrically stimulated in the innervated isolated rabbit heart preparation (*n* = 30). Heart rate (HR), effective refractory period (ERP), ventricular fibrillation threshold (VFT) and electrical restitution were measured as well as NO release from the left ventricle.

**Results:** High voltage with low frequency VNS resulted in the most significant reduction in HR (by −20.6 ± 3.3%, −25.7 ± 3.0% and −30.5 ± 3.0% at 0.1, 1 and 2 ms pulse widths, with minimal increase in NO release. Low voltage and high frequency VNS significantly altered NO release in the left ventricle, whilst significantly flattening the slope of restitution and significantly increasing VFT. HR changes however using low voltage, high frequency VNS were minimal at 20Hz (to 138.5 ± 7.7 bpm (−7.3 ± 2.0%) at 1 ms pulse width and 141.1 ± 6.6 bpm (−4.4 ± 1.1%) at 2 ms pulse width).

**Conclusion:** The protective effects of the VNS are independent of HR reductions demonstrating the likelihood of such effects being as a result of the modulation of more than one molecular pathway. Altering the parameters of VNS impacts neural fibre recruitment in the ventricle; influencing changes in ventricular electrophysiology, the protective effect of VNS against VF and the release of NO from the left ventricle.

## Introduction

Autonomic activity has been shown to be abnormal in cardiac diseases where mortality is high, including in patients with previous myocardial infarction (MI) and heart failure (HF) (Ng, 2016). This abnormal cardiac autonomic activity includes increased sympathetic activity and impaired parasympathetic control and is often an active and adverse contributor to pathogenesis and disease progression (Olshansky et al., 2008; Lymperopoulos et al., 2013). There is strong evidence that the relationship between impaired autonomic control and mortality is due to an increased susceptibility to fatal arrhythmias including ventricular fibrillation (VF), leading to sudden cardiac death (SCD): a complex and unresolved clinical problem.

The fibres of the vagus nerve can be classified as myelinated A and B fibres and unmyelinated C fibres, all of which demonstrate differing activation properties. It is well accepted that the vagus nerve slows heart rate (HR), atrioventricular conduction and decreases atrial contraction, with vagus nerve stimulation (VNS) resulting in anti-arrhythmic effects and acting in a cardioprotective manner (Brack et al., 2013). The mechanisms underlying the anti-arrhythmic properties of VNS on the ventricle however, remain poorly understood. For many decades, it has been known that VNS increases the fibrillation threshold, therefore reducing susceptibility of the heart to VF (Einbrodt, 1895). This effect has since been confirmed in animal studies (Brack et al., 2007a; Ng et al., 2007), where the anti-fibrillatory effect of VNS was shown to be associated with a change in the electrical restitution properties of the heart (Ng et al., 2007) and with an important study by Vanoli et al. (1991) showing a significant prevention of ischaemia-induced VF by vagal stimulation in a conscious animal model of sudden ischaemic cardiac death.

Until recently, it was thought that vagal post-ganglionic nerve terminals modulated cardiac function *via* acetylcholine (ACh) acting on muscarinic receptors. Evidence has since demonstrated that VNS leads to the release of nitric oxide (NO) in the cardiac ventricle, with the anti-arrhythmic properties of the vagus working *via* NO-dependent mechanisms independent of muscarinic receptor activation (Brack et al., 2007a; Brack et al., 2009; Brack et al., 2011). However, the actions of NO on the modulation of cardiac function as a result of VNS are complex and the effect of altering vagal stimulation parameters on NO release in the cardiac ventricle is not well understood.

Over the past decades, research into the potential use of VNS as a safe and effective long term cardiac therapy for HF and cardiac disease has yielded promising results. Pre-clinical experimental models however, often use vagal stimulation parameters resulting in significant chronotropic changes (Brack et al., 2006; Kawada et al., 2006; Kawada et al., 2008). Recent investigations into the effects of low level vagal stimulation, whereby stimulation parameters were selected such that little or no change in HR or in atrioventricular conduction were seen (Li et al., 2009; Cho et al., 2014; Stavrakis et al., 2015), demonstrated the potential to decrease LV infarct size and reduce VF occurrence (Shinlapawittayatorn et al., 2013), and improve LV systolic function in ischaemic heart failure (Hamann et al., 2013). It is known that in MI and HF, central NO biology is disturbed with myocardial neuronal NO synthase (nNOS) being upregulated, likely as a partial compensatory mechanism (Sabbah, 2011). One proposed mechanism *via* which VNS exerts its cardioprotective effects in HF is through the normalisation of NO signalling pathways, specifically with the calibration of nNOS expression in the myocardium (Sabbah et al., 2011; Kalla et al., 2016).

The focus clinically so far has been on direct electrical stimulation of the vagus nerve, with mixed results. Although the ANTHEM-HF study in India (Premchand et al., 2014; Premchand et al., 2016) showed significant positive outcomes on cardiac function, other major multicentre human trials, NECTAR-HF (Zannad et al., 2014) and INOVATE-HF (Gold et al., 2016), failed to achieve their predicted outcomes. This suggests that the stimulation parameters and protocols (De Ferrari and Schwartz, 2011; Ardell et al., 2017) used pre-clinically may have a significant effect of the recruitment of heterogenous nerve populations, potentially explaining the difficulties in translating pre-clinical research into human trials and highlighting the need for the optimisation of stimulation parameters. The use of varying stimulation parameters is thought to significantly impact the recruitment of vagal fibres, with variation in the type and number of vagal fibres possible. Experimental studies so far have provided inadequate information to aid the choice of clinical stimulation parameters, meaning the choice of parameters used has been somewhat arbitrary. Further study to determine the impact of VNS on neural fibre recruitment and the subsequent electrophysiological effects is therefore warranted.

The implications of the vagus nerve exerting its cardiac protective effect *via* a direct nitrergic action in the ventricle have important physiological implications. Therefore, the primary objective of the present study was to investigate NO release and cardiac electrophysiological effects of electrical stimulation of the vagus nerve at a range of clinically relevant parameters using our isolated rabbit heart preparation (Ng et al., 2001a). Unlike earlier studies, we compared the effects of stimulation at varying stimulation strengths, frequencies and pulse widths. In the present experiments, we measured the effects of stimulation on sinus rate, ventricular fibrillation threshold (VFT) and electrical restitution. In addition, this study was designed to evaluate vagal anti-fibrillatory action and NO activity in the left ventricle during VNS.

## Methods

### Ethical Statement

All procedures were undertaken using Adult male New Zealand White rabbits (*n* = 30, 2.0–2.5 kg) in accordance with the United Kingdom Animals (Scientific Procedures) Act 1986, the Guide for the Care of Use of Laboratory Animals Published by the US National Institutes of Health (NIH Publication No. 85-23, revised 1985) and the European Union Directive on the protection of animals for scientific research (2010/63/EU). Local ethics approval was obtained from the University of Leicester animal welfare review board (AWERB) under the Home Office Project Licence PPL70/8501.

### The Innervated Isolated Heart Preparation

The innervated isolated heart preparation has been described previously (Ng et al., 2001a). In brief, animals were sedated, with anaesthesia maintained using i.v. Propofol administered *via* the ear vein. Animals were ventilated *via* a tube inserted into the trachea using a small animal ventilator (Harvard Apparatus Ltd., Edenbridge, Kent, United Kingdom; O_2_-air mixture, 60 breaths/min) whilst the subclavian vessels were ligated and cut. The right cervical vagus was isolated and animals were heparinised (1000IU,i.v.). Following euthanasia with an overdose of Euthetal (111 mg/kg), the anterior portion of the ribcage was removed to allow the cannulation of the descending aorta and the flushing of the heart with ice-cold Tyrode solution. The vertebral column was then transected at the 12th thoracic vertebra and 1st cervical vertebra.

### Langendorff Perfusion

Hearts were perfused with Tyrode solution containing Na^+^138.0; K^+^4.0; Ca^2+^1.8; Mg^2+^1.0; HCO_3_^−^24.0; H_2_PO_4_^−^0.4; Cl^−^124.0; Glucose11.0 (mM) at a flow rate of 100 ml/min *via* a Gilson minipulse 3 peristaltic pump (Anachem, Luton, United Kingdom). The solution was maintained at 37°C (pH7.4) by continuous bubbling with Carbogen [95%O_2_/5%CO_2_]. A 1 mm ID, 2 mm OD polypropylene catheter (Porlex, Kent, United Kingdom) was inserted through the left ventricular (LV) apex for Thebesian venous effluent drainage. Hearts were instrumented to record left ventricular pressure (LVP) and perfusion pressure (PP).

### Cardiac Electrical Recording and Pacing

Ventricular monophasic action potentials (MAP) were recorded at the apical and basal epicardial surfaces by applying MAP contact electrodes (73-0150, Harvard Apparatus, Kent, United Kingdom) and using a custom made DC-coupled high input impedance differential amplifier (Joint Biomedical Workshop, University of Leicester, United Kingdom).

Ventricular pacing was achieved using a bipolar pacing catheter (ADinstruments Ltd., Chalgrove, United Kingdom) inserted into the right ventricular apex. A stimulus was delivered to the heart at double the diastolic pacing threshold using a constant current stimulator (DS7A; Digitimer, Welwyn Garden City, United Kingdom). A pair of electrodes was inserted into the right atrial appendage for recording atrial electrogram (AE). Electrocardiogram (ECG) was recorded by using another pair of electrodes.

### Pacing Protocols

#### Electrical restitution

Standard APD restitution data was obtained using right ventricular pacing at a cycle length (CL) of 300 ms for 20 S1 drive train beats, followed by an extrastimulus (S2). S2 had an initial CL of 300 ms, decreasing in 10 ms increments until S2 reached 200 ms and then decreasing in 5 ms increments until the effective refractory period (ERP) was reached. The ERP was defined as the longest S1-S2 interval that failed to capture the ventricles (Ng et al., 2007).

MAP duration restitution curves were constructed by plotting S2 MAP duration vs. diastolic interval (DI) as described previously (Chauhan et al., 2018). An exponential curve was calculated [MAPD_90_ = MAPD_90max_ (1—e^−DI/T^)] and fitted using Microcal Origin (v6.1, Origin, San Diego, CA, United States) where T = time constant. The maximum slope of the restitution curve was obtained by measuring the first derivative of the fitted curve.

#### Ventricular Fibrillation Threshold

VFT was obtained with right ventricular pacing using 20 pulses at 300 ms CL followed by a rapid 30 beat train at 30 ms CL. Following a 5s rest period, this was repeated and the current increased in increments of 0.5 mA. VFT was defined as the minimum current required to induce sustained VF.

### Vagal Nerve Stimulation

As described previously (Ng et al., 2001b; Brack et al., 2007b), the right vagus nerve was stimulated using a custom-made bipolar silver electrode connected to a constant voltage square pulse stimulator (SD9, Grass Instruments, Astro-Med Inc., United States).

### Stimulation Protocols

The effects on heart rate during direct vagal stimulation at varying strengths (1–20 V) were observed. Two stimulus outputs were used; the stimulus strength that produced a heart rate equivalent to 80% of the maximal response (high voltage) and the stimulus strength that reduced heart rate by 10% from baseline (low voltage).

Stimulation was carried out at varying pulse widths; 0.1, 1 and 2 ms within three protocols. 1) high voltage at 1, 2, and 3 Hz, 2) low voltage at 10, 20, and 30 Hz and 3) low voltage-low frequency at 1, 2, and 3 Hz. Experimental protocols were performed using different experimental preparations. The order of stimulation parameters used was randomised during each experiment and altered between protocols, with a minimum of 30 s rest allowed between protocols.

### DAF-2 Loading and Measuring of NO Fluorescence

An Optoscan spectrophotometer modular system (Cairn Research Ltd., Faversham, United Kingdom) was used to monitor excitation wavelengths at 490 ± 10 nm (*F*_490_). NO fluorescence was measured using a bifurcated light guide as described previously (Patel et al., 2008; Brack et al., 2009). Hearts were loaded with a bolus injection of 4, 5-diaminofluorescein diacetate (DAF-2 DA) (150–250 μl, 1 μm; Calbiochem c/o Merck, Nottingham, United Kingdom) *via* a cannula inserted into the right carotid artery and signals recorded at 490 ± 10 nm from the epicardium of the LV free wall. Analysis of data collected was completed as described previously (Vanoli et al., 1991), with the mean *F*_490_ level being presented and analysed in this study.

### Experimental Protocols

Following stabilisation of baseline NO fluorescence signals under constant pacing at a cycle length of 300 ms (200 bpm), the effect of RVNS on F490 fluorescence was examined during 20 s of nerve stimulation, with a rest period between stimulation protocols.

### Data Recording and Analysis

Functional responses were recorded using a PowerLab 16 channel system [and digitised at 2 kHz using LabChart Software (ADInstruments Ltd.)].

### Chemicals

Unless stated, all chemicals were purchased from Sigma Aldrich, United Kingdom.

### Statistical Analysis

Data analysis was performed using GraphPad Prism7 software. Statistical comparisons were made two-way ANOVA where appropriate with Bonferroni post-hoc test. Data are presented as mean ± SEM; *p* < 0.05 was considered significant.

## Results

The electrophysiological effects of varying right vagus nerve stimulation (RVNS) parameters were examined in eighteen hearts where HR, ERP, VFT and electrical restitution were measured.

### The Effects of High Voltage RVNS

At stimulation frequencies of 1, 2, and 3 Hz, RVNS was carried out in six hearts at voltages of 6.0 ± 1.8 V, 8.2 ± 1.6 V and 7.7 ± 0.8 V (at 0.1,1 and 2 ms pulse widths respectively).

#### Chronotropic Responses to High Voltage RVNS

High voltage RVNS significantly decreased heart rate at all frequencies investigated. At 2 Hz, RVNS decreased HR from a baseline of 146.8 ± 11.2 bpm to 115.6 ± 5.7 bpm, 117.5 ± 10.3 bpm and 114.4 ± 11.2 bpm (at 0.1, 1, and 2 ms pulse widths). At 3 Hz, RVNS reduced HR in a pulse width dependent manner (Table 1). Comparing the decreases in HR at 3Hz at various pulse widths, stimulation at 0.1 ms produced a decrease to 110.2 ± 7.5 bpm (−20.6 ± 3.3%) compared to 105.3 ± 7.3 bpm (−25.7 ± 3.0%) at 1 ms and 99.6 ± 8.6 bpm (−30.5 ± 3.0%) at 2 ms.

**TABLE 1:** The effects of high voltage and low frequency VNS on HR and VFT. HR changes from baseline (BL) to VNS at 1 (A), 2 (B) and 3 Hz (C) at stimulation pulse widths of 0.1, 1 and 2 ms. VFT changes from baseline (BL) to VNS at 1 (D), 2 (E) and 3 Hz (F) at stimulation pulse widths of 0.1, 1, and 2 ms. Data represent mean ± SEM. *p < 0.05, **p < 0.01, ***p < 0.001, ****p < 0.0001 *vs.* corresponding BL, *n* = 6.

		1Hz			2Hz			3Hz		
		0.1 ms	1.0 ms	2.0 ms	0.1 ms	1.0 ms	2.0 ms	0.1 ms	1.0 ms	2.0 ms
HR (bpm)	BL	140.5 ± 10.4	139.4 ± 9.2	144.0 ± 9.7	137.1 ± 8.9	139.2 ± 9.2	140.0 ± 10.1	139.2 ± 9.1	142.1 ± 8.9	143.2 ± 9.6
	VNS	130.3 ± 9.9	121.8 ± 10.7****	125.9 ± 10.6****	115.6 ± 5.7****	117.5 ± 10.3****	114.4 ± 11.2****	110.2 ± 7.5****	105.3 ± 7.3****	99.6 ± 8.6****
VFT (mA)	BL	2.8 ± 0.4	2.8 ± 0.5	2.8 ± 0.5	3.3 ± 0.5	3.3 ± 0.6	2.9 ± 0.4	3.6 ± 0.4	3.8 ± 0.4	3.9 ± 0.5
	VNS	5.0 ± 1.2*	4.9 ± 0.9*	4.8 ± 0.9*	6.3 ± 1.5***	4.8 ± 0.5	5.3 ± 0.6**	5.0 ± 0.4	4.8 ± 0.6	5.5 ± 0.5

#### The Effects of High Voltage RVNS on Ventricular Fibrillation Threshold

At 1 Hz, high voltage RVNS significantly increased VFT at all pulse widths tested [0.1 ms: 2.8 ± 0.4 mA to 5.0 ± 1.2 mA, (*p* < 0.05), 1 ms: 2.8 ± 0.5 mA to 4.9 ± 0.9 mA, (*p <* 0.05) and 2 ms: 2.8 ± 0.5 mA to 4.8 ± 0.9 mA, (*p* < 0.05)]. Stimulation at 2 Hz resulted in significant increases in VFT at 0.1 ms (*p <* 0.001) and 2 ms (*p <* 0.01) (Table 1).

### The Effects of Low Voltage RVNS

With the aim of minimising chronotropic changes, whilst preserving potential anti-arrhythmic effects, RVNS at fixed frequencies of 5,10, and 20 Hz was carried out in a further six hearts at low voltages of 2.4 ± 0.3 V, 1.6 ± 0.1 V and 1.5 ± 0.1 V (at 0.1,1, and 2 ms pulse widths respectively).

#### Chronotropic Responses to Low Voltage RVNS

Similar to the chronotropic changes measured with high voltage RVNS, low voltage RVNS elicited significant reductions in HR at all frequencies tested with no significant differences in HR reduction between 5, 10, and 20 Hz (Table 2). At 5 Hz, low voltage RVNS significantly decreased HR from a baseline of 147.5 ± 4.7 bpm to 140.5 ± 4.2 bpm (−4.7 ± 1.6%) (1 ms) and 141.6 ± 5.1 bpm (−4.0 ± 1.2%) (2 ms). By comparison, increasing the frequency to 20Hz didn’t result in significantly larger decreases in HR (138.5 ± 7.7 bpm (−7.3 ± 2.0%) at 1 ms and 141.1 ± 6.6 bpm (−4.4 ± 1.1%) at 2 ms).

**TABLE 2:** The effects of low voltage and high frequency VNS on HR and VFT. HR changes from baseline (BL) to VNS at 5 (A), 10 (B) and 20 Hz (C) at stimulation pulse widths of 0.1, 1 and 2 ms. VFT changes from baseline (BL) to VNS at 5 (D), 10 (E) and 20 Hz (F) at stimulation pulse widths of 0.1, 1 and 2 ms. Data represent mean ± SEM. *p < 0.05, **p < 0.01, ***p < 0.001, ****p < 0.0001 *vs.* corresponding BL, *n* = 6.

		5Hz			10Hz			20Hz		
		0.1 ms	1.0 ms	2.0 ms	0.1 ms	1.0 ms	2.0 ms	0.1 ms	1.0 ms	2.0 ms
HR (bpm)	BL	147.2 ± 5.0	147.6 ± 4.7	147.5 ± 4.7	148.4 ± 5.8	148.6 ± 5.6	148.3 ± 4.6	149.8 ± 8.0	149.2 ± 7.1	147.3 ± 5.4
	VNS	141.5 ± 5.2	140.5 ± 4.2 **	141.6 ± 5.1*	139.2 ± 6.0***	142.1 ± 4.7*	140.5 ± 4.6**	143.9 ± 7.4*	138.5 ± 7.7****	141.1 ± 6.6*
VFT (mA)	BL	2.8 ± 0.6	2.6 ± 0.6	2.5 ± 0.5	3.3 ± 0.7	3.1 ± 0.5	3.0 ± 0.6	2.8 ± 0.6	2.9 ± 0.7	3.3 ± 0.5
	VNS	5.1 ± 0.9*	5.0 ± 0.7**	3.7 ± 0.6	5.1 ± 0.7	4.8 ± 1.0	5.3 ± 1.2*	4.3 ± 0.7	4.1 ± 0.6	5.1 ± 0.6

#### The Effects of Low Voltage RVNS on Ventricular Fibrillation Threshold

As shown in Figure 2, low voltage RVNS at 5 Hz elicited significant increases in VFT at 0.1 and 1 ms pulse widths (2.8 ± 0.6 mA to 5.1 ± 0.9 mA (*p* < 0.05) and 2.6 ± 0.6 mA to 5.0 ± 0.7 mA (*p* < 0.01) respectively). At subsequent stimulation at 10 Hz, VFT was significantly increased at 2 ms pulse width from 3.0 ± 0.6 mA to 5.3 ± 1.2 mA (*p <* 0.05) (91.0 ± 38.6%) (Table 2).

### The Effects of Low Voltage, Low Frequency RVNS

To investigate the effects of low voltage and low frequency RVNS, electrophysiological effects were measured at stimulation frequencies of 1, 2 and 3 Hz in a total of six hearts at low voltages of 3.3 ± 0.7 V, 1.7 ± 0.3 V, and 1.4 ± 0.2 V at 0.1, 1, and 2 ms pulse widths respectively.

#### Chronotropic Responses to Low Voltage, Low Frequency RVNS

Low voltage, low frequency RVNS produced the smallest percentage changes in HR when compared to the previous two protocols investigated. At 1 and 2 Hz, the majority of HR reductions were small (Table 3). At a frequency of 3 Hz however, RVNS significantly reduced HR from a baseline of 150.1 ± 7.5 bpm to 141.6 ± 5.5 bpm (−5.4 ± 1.2%), 142.3 ± 7.5 bpm (−5.0 ± 1.9%) and 143.1 ± 8.4 bpm (−5.2 ± 1.0%) at pulse widths of 0.1, 1, and 2 ms respectively.

**TABLE 3:** The effects of low voltage and low frequency VNS on HR and VFT. HR changes from baseline (BL) to VNS at 1 (A), 2 (B) and 3 Hz (C) at stimulation pulse widths of 0.1, 1 and 2 ms. VFT changes from baseline (BL) to VNS at 1 (D), 2 (E) and 3 Hz (F) at stimulation pulse widths of 0.1, 1 and 2 ms. Data represent mean ± SEM. *p < 0.05, **p < 0.01, ***p < 0.001 *vs.* corresponding BL, *n* = 6.

		1Hz			2Hz			3Hz		
		0.1 ms	1.0 ms	2.0 ms	0.1 ms	1.0 ms	2.0 ms	0.1 ms	1.0 ms	2.0 ms
HR (bpm)	BL	152.6 ± 9.2	152.5 ± 9.3	151.8 ± 9.0	150.2 ± 7.7	152.2 ± 8.9	153.7 ± 10.2	150.1 ± 7.5	150.4 ± 9.8	150.9 ± 8.5
	VNS	149.6 ± 8.9	148.5 ± 9.0	149.2 ± 9.3	144.5 ± 8.3	146.8 ± 8.8	145.1 ± 9.5***	141.6 ± 5.5***	142.3 ± 7.5**	143.1 ± 8.4**
VFT (mA)	BL	4.3 ± 0.8	4.3 ± 0.8	4.3 ± 0.8	4.3 ± 0.6	4.3 ± 0.6	4.3 ± 0.6	5.2 ± 0.9	4.8 ± 0.8	4.8 ± 0.8
	VNS	6.2 ± 0.9	6.3 ± 1.0	5.9 ± 0.5	6.5 ± 0.8*	6.8 ± 0.9**	6.8 ± 0.9**	7.9 ± 1.0**	7.8 ± 1.2***	6.6 ± 0.9

#### The Effects of Low Voltage, Low Frequency RVNS on Ventricular Fibrillation Threshold

Low voltage, low frequency stimulation at 1 Hz resulted in slight increases in VFT at all pulse widths examined (Table 3) whilst stimulation at a frequency of 2 Hz significantly increased VFT at pulse widths of 0.1 ms (54.6 ± 20.1%), 1 ms (65.9 ± 19.3% and 2 ms (59.8 ± 17.6%). Increasing the frequency of stimulation to 3 Hz resulted in additional increases in VFT (0.1 ms: 5.2 ± 0.9 mA to 7.9 ± 1.0 mA and 1 ms: 4.8 ± 0.8 mA to 7.8 ± 1.2 mA), however they were not significantly different from results measured at 2 Hz.

### The Effect of Varying VNS Stimulation Parameters on Ventricular ERP and Restitution

#### The Effects of High Voltage RVNS on ERP and MAPDR

**FIGURE 1:**
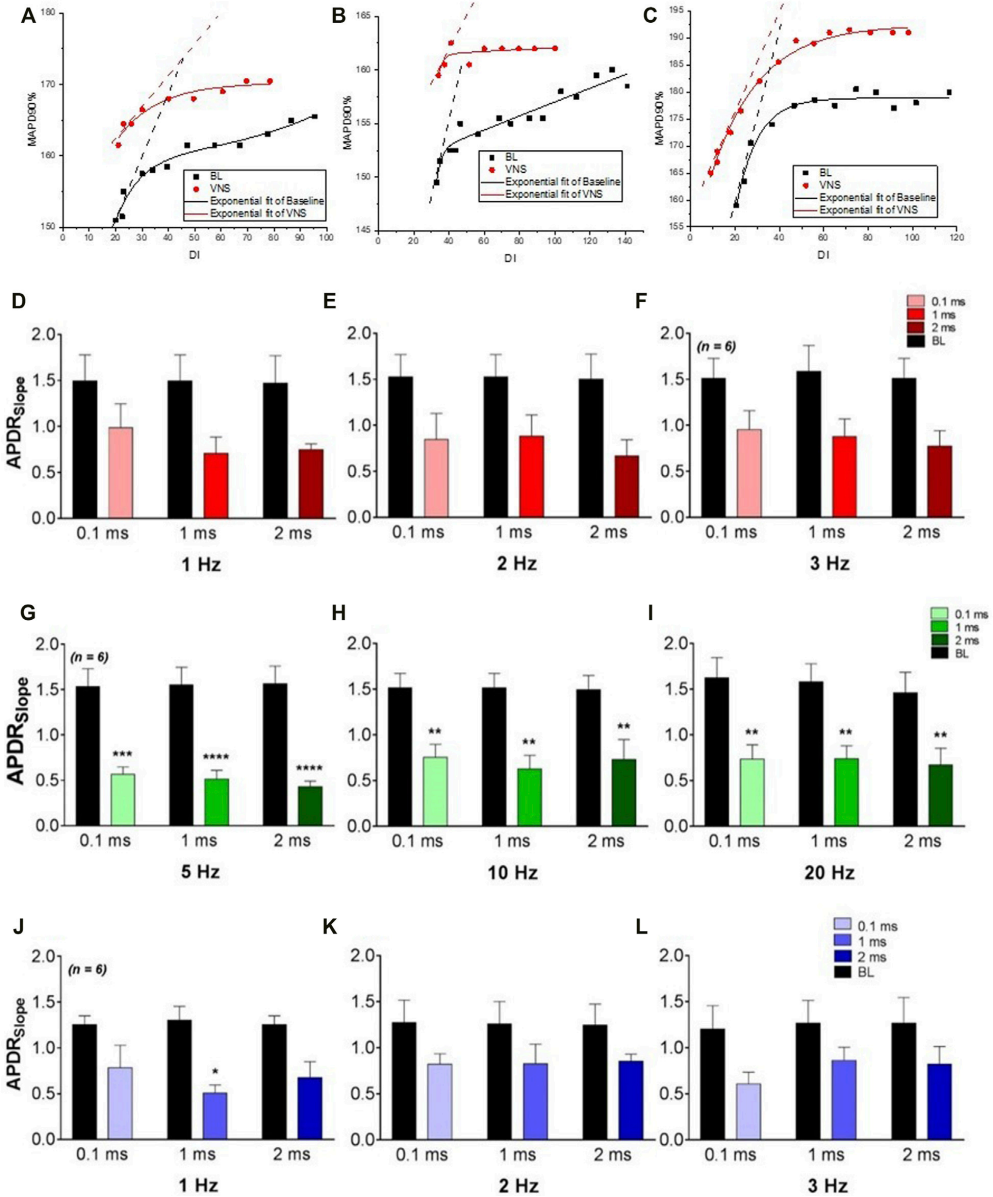
The effects of varying VNS on the maximum slope of restitution. **(A–C)**, plots of APD restitution curves from a typical experiment at baseline and during high voltage **(A)**, low voltage **(B)** and low voltage-low frequency **(C)** VNS. The maximum slope of restitution at baseline (BL) and during high voltage **(D–F)**, low voltage **(G–I)** and low voltage-low frequency **(J–L)** VNS at pulse widths of 0.1 m 1 and 2 ms. Data represent mean ± SEM. *p < 0.05, **p < 0.01, ***p < 0.001, ****p < 0.0001 *vs.* corresponding BL, *n* = 6.

High voltage stimulation at 1 Hz resulted in no significant change in ERP. At an increased frequency of 2 Hz, ERP was significantly prolonged at 0.1 ms (148.3 ± 7.6 ms to 156.7 ± 7.6 ms, *p* < 0.01) and 1 ms (148.3 ± 7.6 ms to 156.7 ± 7.6 ms, *p* < 0.01). At a frequency of 3 Hz, ERP was significantly prolonged at all pulse widths investigated (0.1 ms: 149.2 ± 6.9 ms to 160.0 ± 6.1 ms, *p* < 0.0001, 1 ms PW: 149.2 ± 6.9 ms to 157.5 ± 6.7 ms, *p <* 0.01 and 2 ms: 149.2 ± 6.9 ms to 155.8 ± 7.0 ms, *p* < 0.05). Restitution curves of the MAP duration at corresponding DIs, from the apical and basal epicardium were plotted during baseline and RVNS. At all stimulation parameters, RVNS flattened the maximum slope of restitution (Figures 1E–G) with stimulation at 3Hz, 0.1 ms PW resulting in a significant flattening of the maximum slope of restitution at the apical region (1.5 ± 0.1 to 0.8 ± 0.2, *p* < 0.001). High voltage RVNS caused a larger overall decrease in the maximum slope of restitution at the base compared to the apex.

#### The Effects of Low Voltage RVNS on ERP and MAPDR

Low voltage stimulation resulted in a slight albeit non-significant prolongation of ERP at all frequencies and pulse widths investigated. Low voltage RVNS however, resulted in a significant flattening of the maximum slope of restitution at the both the apex and base at all frequencies examined (Figures 1G–I). Most significantly, RVNS at 0.1, 1, and 2 ms pulse widths at a fixed frequency of 5 Hz, resulted in significant reductions in the maximum slope of restitution (1.5 ± 02 to 0.6 ± 0.1, 1.6 ± 0.2 to 0.5 ± 0.1 and 1.6 ± 0.2 to 0.4 ± 0.1) at the basal epicardium. At all stimulation parameters, low voltage RVNS was shown to have a larger overall effect at decreasing the maximum slope of restitution at the base compared to the apex, with the maximum change at apex being −51.9 ± 0.8.1% compared to −69.7 ± 6.0% at base.

#### The Effects of Low Voltage, Low Frequency RVNS on ERP and MAPDR

Low voltage, low frequency RVNS elicited small changes in ERP at all stimulation parameters, with larger increases in ERP at 3Hz compared to 1 and 2 Hz. At 3 Hz, significant prolongation occurred at both 0.1 and 1 ms pulse widths [144.2 ± 3.5 ms to 149.2 ± 3.0 ms (*p* < 0.05) and 144.2 ± 3.5 ms to 150.0 ± 3.4 ms (*p* < 0.01)]. The effects of low voltage, low frequency on the maximum slope of restitution at both apex and base were measured. At all stimulation parameters investigated, RVNS flattened the slope, however no significant changes were noted (Figures 1J–L). Unlike with previous protocols, no significant difference was identified between the maximum slope of restitution at the apex and base.

### The Effect of RVNS on Ventricular NO Release

**FIGURE 2:**
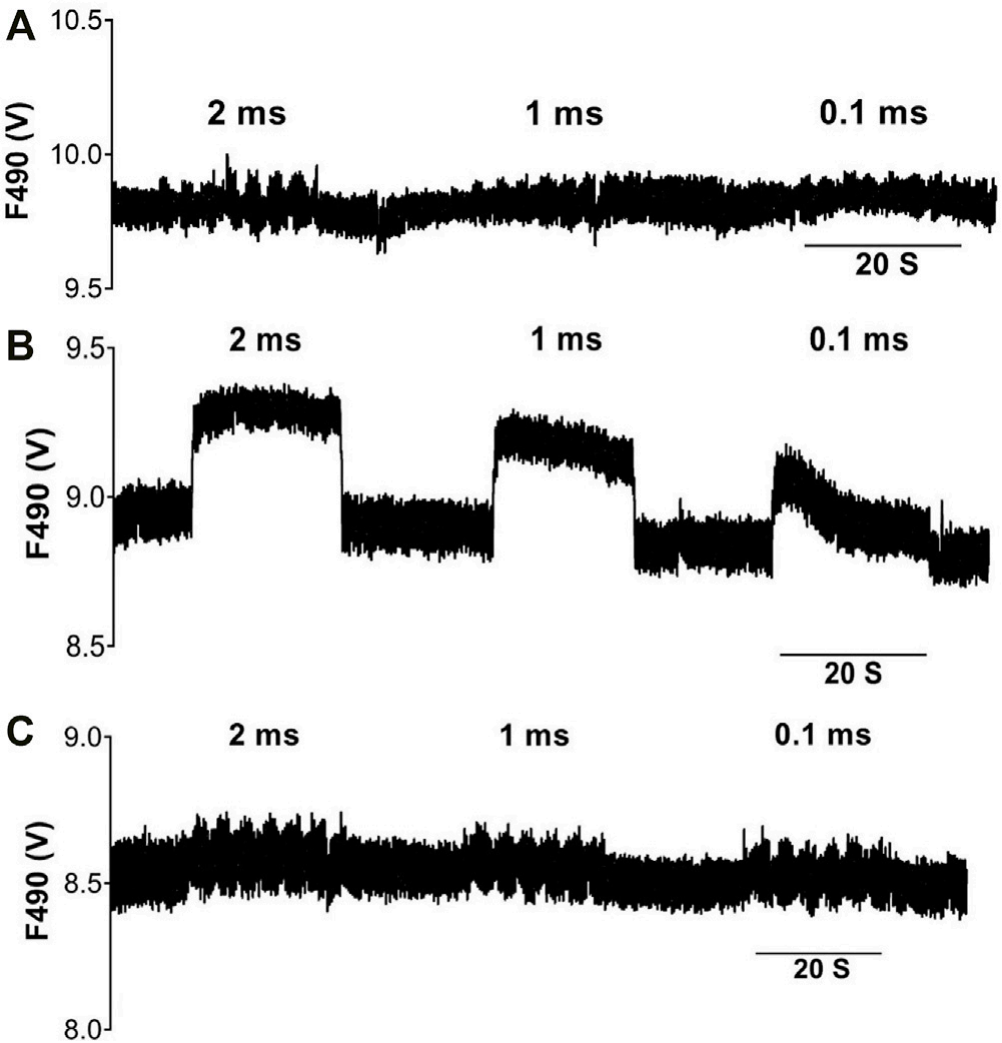
Voltage and frequency dependent changes in NO fluorescence during VNS. Raw data illustrating DAF-2 fluorescence at a 490 nm (F490) excitation wavelength during high voltage-low frequency (3 Hz) stimulation **(A)**, low voltage-high frequency (20 Hz) stimulation **(B)** and low voltage-low frequency (3 Hz) stimulation **(C)**, at varying pulse widths.

**FIGURE 3:**
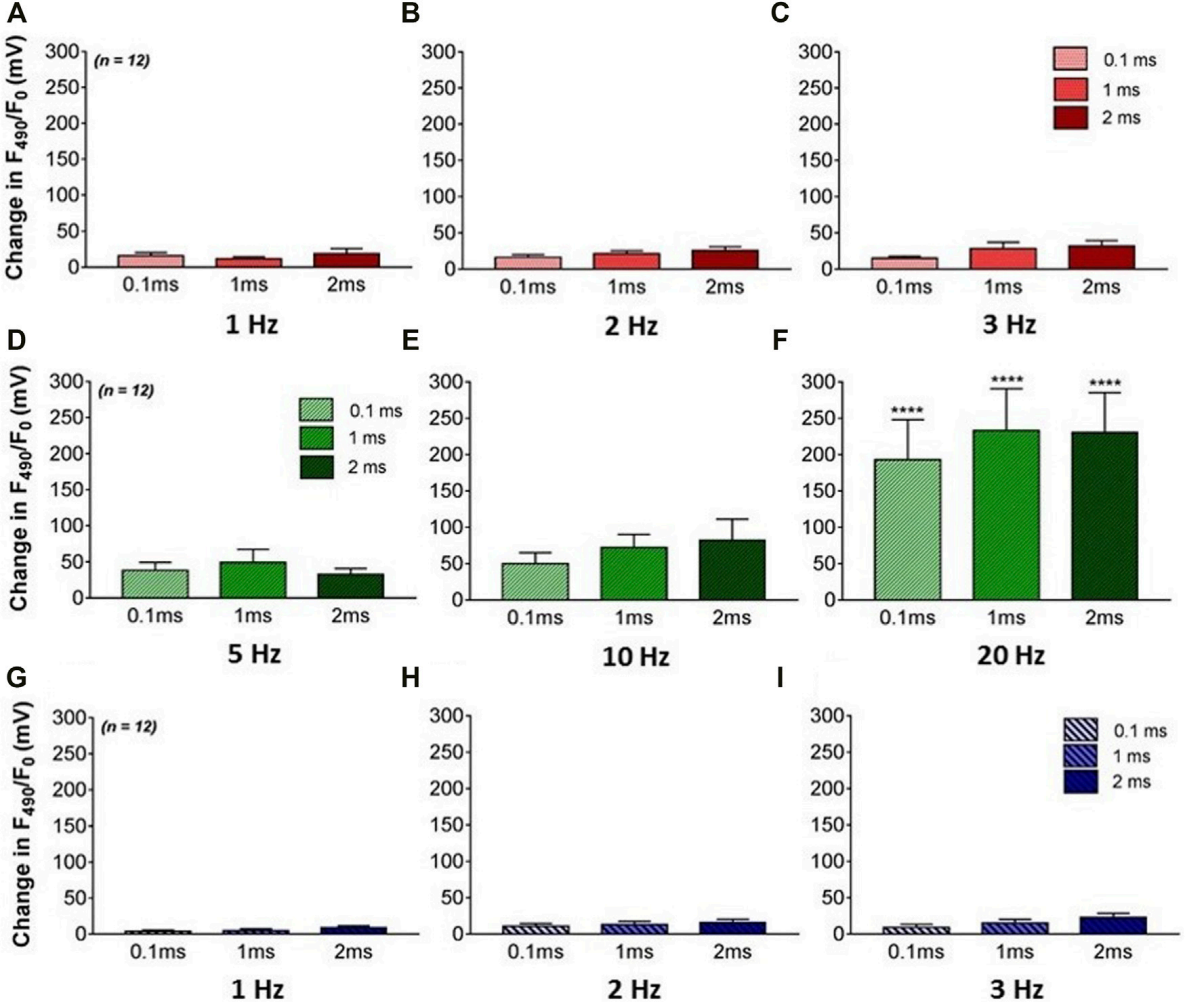
The effects of VNS on NO release in the left ventricle. **(A–C)** Mean F490 levels during high voltage and low frequency stimulation at 1 **(A)**, 2 **(B)** and 3 Hz **(C)** VNS at 0.1, 1, and 2 ms pulse widths. **(D–F)** Mean F490 levels during low voltage and high frequency stimulation at 5 **(D)**, 10 **(E)** and 20Hz **(F)** VNS at 0.1, 1, and 2 ms pulse widths. **(G–I)** Mean F490 levels during low voltage and low frequency stimulation at 1 **(G)**, 2 **(H)** and 3 Hz **(I)** VNS at 0.1, 1, and 2 ms pulse widths. Data represented as mean ± SEM. ****p < 0.0001 *vs.* corresponding BL *n* = 12.

The effects of varying VNS stimulation parameters on NO release in the left ventricle were examined in twelve hearts (Figure 2). Altering the pulse width of stimulation showed no significant change in NOFL during all three stimulation protocols. High voltage VNS resulted in increases of NOFL ranging from 11.6 ± 2.9mV to 31.9 ± 7.5 mV (Figures 3A–C). There were significant increases in NOFL from baseline during low voltage stimulation at a frequency of 20 Hz at all pulse widths examined (*p* < 0.0001) (Figure 3F). Low frequency, low voltage stimulation resulted in only small increases in NOFL (4.2 ± 1.5 mV to 22.7 ± 5.9 mV).

## Discussion

To the best of our knowledge, this is the first study to investigate the effects of clinically relevant vagus nerve stimulation parameters on cardiac electrophysiology and NO release in the ventricle, in rabbit hearts. Furthermore, these experiments were performed using an isolated innervated heart preparation to avoid the influence of extrinsic circulatory and neurohumoral factors.

Using electrical stimulation of the cervical portion of the vagus nerve, the results indicate that the protective, anti-arrhythmic effects of VNS demonstrated previously (Vanoli et al., 1991; Ng et al., 2007; Huang et al., 2015; Zhang et al., 2016) are observed both with and without reductions in HR. VNS at parameters capable of producing significant changes in HR is thought to occur *via* muscarinic receptor activation at the sinus node (Woolley et al., 1987; Vanoli et al., 1991; Herring and Paterson, 2001). The effects shown in this study at high stimulation strengths, reinforce the conclusions of such studies.

In addition however, this study has examined the effects of low vagal stimulation strength, resulting in only small HR reductions, on ventricular electrophysiology and the anti-fibrillatory actions of the vagus. At levels where small heart rate reductions were noted, similar levels of anti-arrhythmic protection shown by increases in VFT and ERP and reduction in the maximal slope of electrical restitution were exhibited. This indicates that vagal protection occurs independently of HR reduction (Vanoli et al., 1991; Shinlapawittayatorn et al., 2013), which appears most likely to be as a result of the modulation of an alternative molecular pathway.

The results of the present study indicate that the vagus nerve is composed of functionally distinct groups of fibres that selectively target discrete regions of the heart. Early misconceptions regarding the composition of the vagus and the presence of cardiotropic sympathetic efferent fibres innervating the heart as well as the apparent lack or sparsity of cardiac parasympathetic postganglionic in the ventricle, have since been invalidated (Coote, 2013). Stimulation of the vagus nerve in this study would therefore have led to the recruitment of parasympathetic efferent nerve fibres (Kalla et al., 2016); believed to deliver basal parasympathetic tone to the heart (Yamakawa et al., 2015) and directly act on ventricular myocytes (Coote, 2013).

**FIGURE 4:**
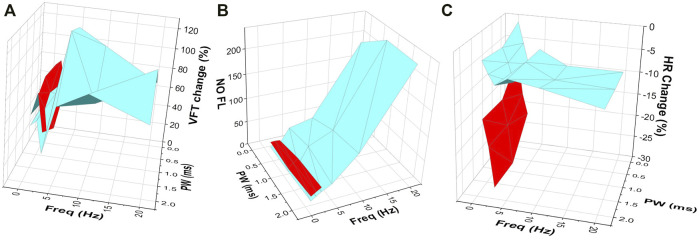
The effects of VNS at varying stimulation parameters on **(A)** VFT **(B)** NO fluorescence and **(C)** HR change. Three-dimensional plots illustrating the effects of varying stimulation parameters on changes in VFT, NO fluorescence and heart rate, highlighting the important effect of VNS at high voltages and low frequencies (red) on heart rate **(C)**, with low voltage and high frequency stimulation (blue) producing a more prominent effect on VFT **(A)** and NO release in the ventricle **(B)**. Data in red represents high voltage stimulation. Data in blue represents low voltage stimulation.

High voltage in conjunction with a low frequency of stimulation, resulted in the most significant changes in HR, ERP, VFT, and maximum slope of restitution (Figure 4). The similarity of these data appears to strengthen the conclusions of prior studies where similar stimulation parameters evoked significant protective effects (Ng et al., 2007; Brack et al., 2011). This is likely to be due to increased recruitment of fibres known to be present within the vagal nerves. At this level of stimulation, thick myelinated or A fibres, thin myelinated fibres or B fibres along with and non-myelinated or C fibres are likely to evoke the cardiac effect noted (Woolley et al., 1987; Tosato et al., 2006).

By comparison, stimulation at low voltages with high frequencies resulted in only smaller HR changes however, these changes were accompanied by more robust increases in VFT (Figure 4) and reductions in the maximum slope of restitution, reiterating the HR independent cardio-protective effects of low level VNS previously reported (Katare et al., 2009) and supporting the notion that VNS leads to anti-arrhythmic effects in the cardiac ventricle, independent of muscarinic receptor activation. It is likely that VNS at the lower stimulation parameters investigated here, resulted in the reduced recruitment of vagal B fibres, with the majority of effects occurring as a result of the recruitment of thick myelinated A fibres (Tosato et al., 2006).

Using electrical stimulation applied at varying pulse widths, the results indicate that at shorter pulse widths, a larger stimulus strength was required in order to produce a similar reduction in HR when compared to longer pulse widths. In addition, altering pulse width appeared to have little impact on changes in ventricular electrophysiology. However, due to the nature of the protocols used where stimulation voltages were determined based on HR changes, the effects of altering pulse width could not be conclusively determined and warrants further investigation.

It is widely accepted that NO is involved in aspects of vagal control of cardiac function. Direct testing of the possibility that NO has a role in the ventricular effects of VNS in the rabbit demonstrated the anti-arrhythmic effects of nNOS-derived NO (Brack et al., 2011; Stavrakis et al., 2016), in line with experiments previously conducted in a porcine model (Kumar et al., 2003). Brack et al. (2011) demonstrated an increase in VFT and a reduction in the slope of restitution during VNS, which was preserved during muscarinic blockade, suggesting the role of independent mechanisms for acetylcholine and NO release *via* different populations of efferent parasympathetic fibres. It is feasible therefore to assume that the increase in VFT seen in this study would remain during muscarinic blockade.

The release of NO seen in the ventricle in this study appears dependent upon the level of vagal stimulation, with significant increases in NO evident during low voltage and high frequency stimulation (Figure 3). Significant levels of ventricular NO release were accompanied by larger increases in VFT and reductions in the slope of restitution when compared to stimulation at lower frequencies, suggesting NO release due to VNS is frequency dependent, in line with previous data (Brack et al., 2009). In contrast, the data in this study shows VNS at higher voltages appears to result in larger chronotropic effects, potentially as a result of VNS acting at the atrial level *via* muscarinic receptor activation and further outlining the likelihood of independent mechanisms of action dependent upon the population of efferent parasympathetic fibres being recruited during stimulation of the vagus.

Despite this, our study has demonstrated further the complexity of ventricular arrhythmias and although we know NO plays a key role, this appears entirely dependent upon the level and strength of vagal stimulation, and warrants further investigation to determine the precise mechanisms at play along with the precise pathway through which cervical vagal fibres lead to the release of NO in the ventricle.

### Clinical Implications

It is well recognised that an increased susceptibility to fatal arrhythmias occurs in diseases such as heart failure and following myocardial infarction, often due to an imbalance in the autonomic control of cardiac function. Over the past decade, the previously underappreciated importance of vagal nerve stimulation as a therapeutic target has become more widely recognised. Early phase research has been conducted to investigate the potential effects of non-invasive stimulation of the vagus nerve *via* transcutaneous electrical nerve stimulation (TENS) (Clancy et al., 2014) however, the focus of larger scale clinical studies was on direct electrical stimulation of the vagus nerve using implantable devices (Zannad et al., 2014; Gold et al., 2016). Unfortunately such studies have so far failed to show significant improvements in clinical outcomes, most likely due to the stimulation parameters used. The ANTHEM-HF study (Premchand et al., 2014; Premchand et al., 2016) showed significant positive outcomes on cardiac function, notably with some degree of heart rate change evident during stimulation whilst NECTAR-HF (Zannad et al., 2014) and INOVATE-HF (Gold et al., 2016), failed to achieve their predicted outcomes where inadequate recruitment of relevant vagal fibres could have been the underlying reason. Despite this, early clinical research has been promising, showing clinical improvement and evidencing the anti-arrhythmic effects of vagal nerve stimulation. In accord, the present study demonstrates that even at low voltage of vagus stimulation with high frequency resulting in small reductions in HR, significant cardiac protective effects are evident, correlating with and expanding previous findings in experimental models (Shinlapawittayatorn et al., 2013; Cho et al., 2014; Stavrakis et al., 2015) and highlighting the importance of using the correct combination of stimulation parameters to produce the desired cardiac effect. This data suggests that it is not solely the number of vagal fibres being recruited but the type and frequency at which these fibres are stimulated which are crucial in producing a successful therapeutic outcome. The data in this study also highlights the further need to identify the precise mechanisms of the parasympathetic pathway at the level of the myocardium in order to delineate a more focused method of targeting vagal stimulation.

### Limitations

In using the rabbit isolated innervated heart preparation, it is possible to interpret the direct functional effects of cervical VNS on ventricular electrophysiology, in the absence of confounding external factors. Caution is also warranted in the extrapolation of such data to humans, with differences between the results of this study and human clinical trial data being explained by such. The similarity however of rabbit cardiac and neural anatomy and function with that of other mammals (Janig, 2006) as well as humans enables us to draw the conclusions shown. Stimulation of the vagus nerve produced responses in line with those shown previously (Vanoli et al., 1991; Premchand et al., 2014), further supporting the use of the innervated preparation in this study. It is also important to acknowledge that the use of the isolated innervated heart preparation simplifies the neuronal activity potentially seen *in vivo*. Study of the protective mechanisms of vagal nerve stimulation in an *in vivo* experimental model would inform of the true neuronal hierarchy, feedback mechanisms and interactions involved in altering cardiac electrophysiology and inducing anti-arrhythmic properties.

The direct anatomical evidence of nitrergic neurons in the ventricle is limited (Pauza et al., 2013; Pauziene et al., 2016; Wake and Brack, 2016), however a recent study by Allen et al. (2018), showed a clear demonstration of a heterogenous network of nerves and neurons immunoreactive solely for neuronal nitric oxide synthase (nNOS) in the rabbit ventricle and providing anatomical evidence supporting the hypothesis that the anti-fibrillatory effect of the cervical VNS is dependent upon niteregic postganglionic fibres. We do however acknowledge that the use of a bifurcated single light guide system to measure NO release in this study however, provides only a snapshot of the NO activity in the left ventricle. Despite this, the data obtained expands on previous results (Brack et al., 2007a; Brack et al., 2009) and demonstrates the protective effects of VNS against ventricular arrhythmia is associated with the release of NO even at clinically relevant stimulation parameters.

## Conclusion

In summary, the present study tested the effects of altering vagus nerve stimulation strengths and frequencies on cardiac electrophysiology and function. Altering these parameters impacts neural fibre recruitment and therefore influences changes in ventricular electrophysiology, the protective effect of VNS against VF and also the release of NO from the left ventricle. The protective effects of the VNS are independent of HR reductions (which are likely to represent the effects of cholinergic pathways), compared to the frequency dependent changes in VFT as a result of nitrergic pathways. This study provides an important insight into VNS and improving the use of vagal stimulation devices as a therapeutic mechanism in cardiac disease and identifies the need for further evaluation of the post-ganglionic pathways in the ventricle activated by VNS.

## Data Availability

The original contributions presented in the study are included in the article/supplementary material, further inquiries can be directed to the corresponding author.

## References

[B1] Allen E. Coote J. H. Grubb B. D. Batten T. F. C. Pauza D. H. Ng G. A. (2018). Electrophysiological Effects of Nicotinic and Electrical Stimulation of Intrinsic Cardiac Ganglia in the Absence of Extrinsic Autonomic Nerves in the Rabbit Heart. Heart Rhythm 15, 1698–1707. 10.1016/j.hrthm.2018.05.018 29800749 PMC6207532

[B2] Ardell J. L. Nier H. Hammer M. Southerland E. M. Ardell C. L. Beaumont E. (2017). Defining the Neural Fulcrum for Chronic Vagus Nerve Stimulation: Implications for Integrated Cardiac Control. J. Physiol. 595 (22), 6887–6903. 10.1113/jp274678 28862330 PMC5685838

[B3] Brack K. E. Patel V. H. Coote J. H. Ng G. A. (2007). Nitric Oxide Mediates the Vagal Protective Effect on Ventricular Fibrillation via Effects on Action Potential Duration Restitution in the Rabbit Heart. J. Physiol. 583 (Pt 2), 695–704. 10.1113/jphysiol.2007.138461 17627986 PMC2277035

[B4] Brack K. E. Coote J. H. Ng G. A. (2006). The Effect of Direct Autonomic Nerve Stimulation on Left Ventricular Force in the Isolated Innervated Langendorff Perfused Rabbit Heart. Aut. Neurosci. 124, 69–80. 10.1016/j.autneu.2005.11.005 16455307

[B5] Brack K. E. Coote J. H. Ng G. A. (2011). Vagus Nerve Stimulation Protects against Ventricular Fibrillation Independent of Muscarinic Receptor Activation. Cardiovasc. Res. 91, 437–446. 10.1093/cvr/cvr105 21576131

[B6] Brack K. E. Patel V. H. Coote J. H. Ng G. A. (2007). Nitric Oxide Mediates the Vagal Protective Effect on Ventricular Fibrillation via Effects on Action Potential Duration Restitution in the Rabbit Heart. J. Physiol. 583, 695–704. 10.1113/jphysiol.2007.138461 17627986 PMC2277035

[B7] Brack K. E. Patel V. H. Mantravardi R. Coote J. H. Ng G. A. (2009). Direct Evidence of Nitric Oxide Release from Neuronal Nitric Oxide Synthase Activation in the Left Ventricle as a Result of Cervical Vagus Nerve Stimulation. J. Physiol. 587, 3045–3054. 10.1113/jphysiol.2009.169417 19403619 PMC2718260

[B8] Brack K. E. Winter J. Ng G. A. (2013). Mechanisms Underlying the Autonomic Modulation of Ventricular Fibrillation Initiation-Tentative Prophylactic Properties of Vagus Nerve Stimulation on Malignant Arrhythmias in Heart Failure. Heart Fail Rev. 18 (4), 389–408. 10.1007/s10741-012-9314-2 22678767 PMC3677978

[B9] Chauhan R. A. Coote J. Allen E. Pongpaopattanakul P. Brack K. E. Ng G. A. (2018). Functional Selectivity of Cardiac Preganglionic Sympathetic Neurones in the Rabbit Heart. Int. J. Cardiol. 264 (264), 70–78. 10.1016/j.ijcard.2018.03.119 29657079 PMC5968349

[B10] Cho Y. Cha M.-J. Choi E.-K. Oh I.-Y. Oh S. (2014). Effects of Low-Intensity Autonomic Nerve Stimulation on Atrial Electrophysiology. Korean Circ. J. 44, 243–249. 10.4070/kcj.2014.44.4.243 25089136 PMC4117845

[B11] Clancy J. A. Mary D. A. Witte K. K. Greenwood J. P. Deuchars S. A. Deuchars J. (2014). Non-invasive Vagus Nerve Stimulation in Healthy Humans Reduces Sympathetic Nerve Activity. Brain Stimul. 7, 871–877. 10.1016/j.brs.2014.07.031 25164906

[B12] Coote J. H. (2013). Myths and Realities of the Cardiac Vagus. J. Physiology 591, 4073–4085. 10.1113/jphysiol.2013.257758 PMC3779103 23878363

[B13] De Ferrari G. M. Schwartz P. J. (2011). Vagus Nerve Stimulation: from Pre-clinical to Clinical Application: Challenges and Future Directions. Heart Fail Rev. 16 (2), 195–203. 10.1007/s10741-010-9216-0 21165697

[B14] Einbrodt (1895). Uber herzeizung und ihr verhaeltins zum blutdruck. Vienna: Akademie der Wissenschaften, 345–359.

[B15] Gold M. R. Van Veldhuisen D. J. Hauptman P. J. Borggrefe M. Kubo S. H. Lieberman R. A. (2016). Vagus Nerve Stimulation for the Treatment of Heart Failure. J. Am. Coll. Cardiol. 68 (2), 149–158. 10.1016/j.jacc.2016.03.525 27058909

[B16] Hamann J. J. Ruble S. B. Stolen C. Wang M. Gupta R. C. Rastogi S. (2013). Vagus Nerve Stimulation Improves Left Ventricular Function in a Canine Model of Chronic Heart Failure. Eur. J. Heart Fail. 15 (12), 1319–1326. 10.1093/eurjhf/hft118 23883651 PMC3895958

[B17] Herring N. Paterson D. J. (2001). Nitric oxide‐cGMP Pathway Facilitates Acetylcholine Release and Bradycardia during Vagal Nerve Stimulation in the guinea‐pigin Vitro. J. Physiology 535, 507–518. 10.1111/j.1469-7793.2001.00507.x PMC2278790 11533140

[B18] Huang J. Qian J. Yao W. Wang N. Zhang Z. Cao C. (2015). Vagus Nerve Stimulation Reverses Ventricular Electrophysiological Changes Induced by Hypersympathetic Nerve Activity. Exp. Physiol. 100, 239–248. 10.1113/expphysiol.2014.082842 25720663

[B19] Janig W. (2006). The Integrative Action of the Autonomic Nervous System: Neurobiology of Homeostasis. New York: Cambridge University Press, 1–610.

[B20] Kalla M. Chotalia M. Coughlan C. Hao G. Crabtree M. J. Tomek J. (2016). Protection against Ventricular Fibrillation via Cholinergic Receptor Stimulation and the Generation of Nitric Oxide. J. Physiol. 594, 3981–3992. 10.1113/JP271588 26752781 PMC4794549

[B21] Katare R. G. Ando M. Kakinuma Y. Arikawa M. Handa T. Yamasaki F. (2009). Vagal Nerve Stimulation Prevents Reperfusion Injury through Inhibition of Opening of Mitochondrial Permeability Transition Pore Independent of the Bradycardiac Effect. J. Thorac. Cardiovasc. Surg. 137, 223–231. 10.1016/j.jtcvs.2008.08.020 19154929

[B22] Kawada T. Yamazaki T. Akiyama T. Kitagawa H. Shimizu S. Mizuno M. (2008). Vagal Stimulation Suppresses Ischemia-Induced Myocardial Interstitial Myoglobin Release. Life Sci. 83, 490–495. 10.1016/j.lfs.2008.07.013 18713640

[B23] Kawada T. Yamazaki T. Akiyama T. Li M. Ariumi H. Mori H. (2006). Vagal Stimulation Suppresses Ischemia-Induced Myocardial Interstitial Norepinephrine Release. Life Sci. 78, 882–887. 10.1016/j.lfs.2005.05.087 16125731

[B24] Kumar K. Nguyen K. Waxman S. Nearing B. D. Wellenius G. A. Zhao S. X. (2003). Potent Antifibrillatory Effects of Intrapericardial Nitroglycerin in the Ischemic Porcine Heart. J. Am. Coll. Cardiol. 41, 1831–1837. 10.1016/s0735-1097(03)00340-1 12767672

[B25] Li S. Scherlag B. J. Yu L. Sheng X. Zhang Y. Ali R. (2009). Low-Level Vagosympathetic Stimulation. Circ Arrhythmia Electrophysiol. 2, 645–651. 10.1161/circep.109.868331 19948505

[B26] Lymperopoulos A. Rengo G. Koch W. J. (2013). Adrenergic Nervous System in Heart Failure. Circ. Res. 113, 739–753. 10.1161/circresaha.113.300308 23989716 PMC3843360

[B27] Ng G. A. Brack K. E. Coote J. H. (2001). Effects of Direct Sympathetic and Vagus Nerve Stimulation on the Physiology of the Whole Heart-Aa Novel Model of Isolated Langendorff Perfused Rabbit Heart with Intact Dual Autonomic Innervation. Exp. Physiol. 86 (3), 319–329. 10.1113/eph8602146 11471534

[B28] Ng G. A. Brack K. E. Coote J. H. (2001). Differential Effects of Left and Right Vagus Nerve Stimulation on Sinoatrial and Atrioventricular Nodes but Not on Ventricular Electrophysiology - Studies in the Isolated Rabbit Heart with Intact Autonomic Innervation. J. Physiol. 531, 182.

[B29] Ng G. A. Brack K. E. Patel V. H. Coote J. H. (2007). Autonomic Modulation of Electrical Restitution, Alternans and Ventricular Fibrillation Initiation in the Isolated Heart. Cardiovasc Res. 73, 750–760. 10.1016/j.cardiores.2006.12.001 17217937

[B30] Ng G. A. (2016). Neuro-cardiac Interaction in Malignant Ventricular Arrhythmia and Sudden Cardiac Death. Aut. Neurosci. 199, 66–79. 10.1016/j.autneu.2016.07.001 27423297

[B31] Olshansky B. Sabbah H. N. Hauptman P. J. Colucci W. S. (2008). Parasympathetic Nervous System and Heart Failure. Circulation 118, 863–871. 10.1161/circulationaha.107.760405 18711023

[B32] Patel V. H. Brack K. E. Coote J. H. Ng G. A. (2008). A Novel Method of Measuring Nitric-oxide-dependent Fluorescence Using 4,5-diaminofluorescein (DAF-2) in the Isolated Langendorff-Perfused Rabbit Heart. Pflugers Arch. - Eur. J. Physiol. 456, 635–645. 10.1007/s00424-007-0440-y 18180949

[B33] Pauza D. H. Saburkina I. Rysevaite K. Inokaitis H. Jokubauskas M. Jalife J. (2013). Neuroanatomy of the Murine Cardiac Conduction System. Aut. Neurosci. 176, 32–47. 10.1016/j.autneu.2013.01.006 23403121

[B34] Pauziene N. Alaburda P. Rysevaite-Kyguoliene K. Pauza A. G. Inokaitis H. Masaityte A. (2016). Innervation of the Rabbit Cardiac Ventricles. J. Anat. 228, 26–46. 10.1111/joa.12400 26510903 PMC4694158

[B35] Premchand R. K. Sharma K. Mittal S. Monteiro R. Dixit S. Libbus I. (2014). Autonomic Regulation Therapy via Left or Right Cervical Vagus Nerve Stimulation in Patients with Chronic Heart Failure: Results of the ANTHEM-HF Trial. J. Cardiac Fail. 20 (11), 808–816. 10.1016/j.cardfail.2014.08.009 25187002

[B36] Premchand R. K. Sharma K. Mittal S. Monteiro R. Dixit S. Libbus I. (2016). Extended Follow-Up of Patients with Heart Failure Receiving Autonomic Regulation Therapy in the ANTHEM-HF Study. J. Cardiac Fail. 22 (8), 639–642. 10.1016/j.cardfail.2015.11.002 26576716

[B37] Sabbah H. N. (2011). Electrical Vagus Nerve Stimulation for the Treatment of Chronic Heart Failure. Cleve Clin. J. Med. 78 Suppl 1 (1), S24–S29. 10.3949/ccjm.78.s1.04 PMC3817894 21972326

[B38] Sabbah H. N. Ilsar I. Zaretsky A. Rastogi S. Wang M. Gupta R. C. (2011). Vagus Nerve Stimulation in Experimental Heart Failure. Heart Fail Rev. 16 (2), 171–178. 10.1007/s10741-010-9209-z 21128115 PMC3784341

[B39] Shinlapawittayatorn K. Chinda K. Palee S. Surinkaew S. Thunsiri K. Weerateerangkul P. (2013). Low-amplitude, Left Vagus Nerve Stimulation Significantly Attenuates Ventricular Dysfunction and Infarct Size through Prevention of Mitochondrial Dysfunction during Acute Ischemia-Reperfusion Injury. Heart Rhythm 10 (11), 1700–1707. 10.1016/j.hrthm.2013.08.009 23933295

[B40] Stavrakis S. Scherlag B. J. Fan Y. Liu Y. Mao J. Varma V. (2016). Inhibition of Atrial Fibrillation by Low-Level Vagus Nerve Stimulation: the Role of the Nitric Oxide Signaling Pathway. J. Interv. Card. Electrophysiol. 36, 199–208. 10.1007/s10840-012-9752-8 23179922

[B41] Stavrakis S. Humphrey M. B. Scherlag B. J. Hu Y. Jackman W. M. Nakagawa H. (2015). Low-level Transcutaneous Electrical Vagus Nerve Stimulation Suppresses Atrial Fibrillation. J. Am. Coll. Cardiol. 65, 867–875. 10.1016/j.jacc.2014.12.026 25744003 PMC4352201

[B42] Tosato M. Yoshida K. Toft E. Nekrasas V. Struijk J. J. (2006). Closed-loop Control of the Heart Rate by Electrical Stimulation of the Vagus Nerve. Med. Bio Eng. Comput. 44, 161–169. 10.1007/s11517-006-0037-1 16937157

[B43] Vanoli E. De Ferrari G. M. Stramba-Badiale M. Hull S. S. Foreman R. D. Schwartz P. J. (1991). Vagal Stimulation and Prevention of Sudden Death in Conscious Dogs with a Healed Myocardial Infarction. Circ. Res. 68, 1471–1481. 10.1161/01.res.68.5.1471 2019002

[B44] Wake E. Brack K. (2016). Characterization of the Intrinsic Cardiac Nervous System. Aut. Neurosci. 199, 3–16. 10.1016/j.autneu.2016.08.006 27568996

[B45] Woolley D. C. McWilliam P. N. Ford T. W. Clarke R. W. (1987). The Effect of Selective Electrical Stimulation of Non-myelinated Vagal Fibres on Heart Rate in the Rabbit. J. Aut. Nerv. Syst. 21, 215–221. 10.1016/0165-1838(87)90024-5 2897392

[B46] Yamakawa K. Rajendran P. S. Takamiya T. Yagishita D. So E. L. Mahajan A. (2015). Vagal Nerve Stimulation Activates Vagal Afferent Fibers that Reduce Cardiac Efferent Parasympathetic Effects. Am. J. Physiology-Heart Circulatory Physiology 309, H1579–H1590. 10.1152/ajpheart.00558.2015 PMC4666973 26371172

[B47] Zannad F. De Ferrari G. M. Tuinenburg A. E. Wright D. Brugada J. Butter C. (2014). Chronic Vagal Stimulation for the Treatment of Low Ejection Fraction Heart Failure: Results of the NEural Cardiac TherApy foR Heart Failure (NECTAR-HF) Randomized Controlled Trial. Eur. Heart J. 36 (7), 425–433. 10.1093/eurheartj/ehu345 25176942 PMC4328197

[B48] Zhang L. Lu Y. Sun J. Zhou X. Tang B. (2016). Subthreshold Vagal Stimulation Suppresses Ventricular Arrhythmia and Inflammatory Response in a Canine Model of Acute Cardiac Ischaemia and Reperfusion. Exp. Physiol. 101, 41–49. 10.1113/ep085518 26553757

